# Critical Assessment of Clean-Up Techniques Employed in Simultaneous Analysis of Persistent Organic Pollutants and Polycyclic Aromatic Hydrocarbons in Fatty Samples

**DOI:** 10.3390/toxics10010012

**Published:** 2022-01-01

**Authors:** Lucie Drábová, Darina Dvořáková, Kateřina Urbancová, Tomáš Gramblička, Jana Hajšlová, Jana Pulkrabová

**Affiliations:** Department of Food Analysis and Nutrition, Faculty of Food and Biochemical Technology, University of Chemistry and Technology, Prague, Technická 3, 166 28 Prague, Czech Republic; Lucie.drabova@vscht.cz (L.D.); darina.lankova@vscht.cz (D.D.); katerina.urbancova@vscht.cz (K.U.); tomas.gramblicka@vscht.cz (T.G.); jana.hajslova@vscht.cz (J.H.)

**Keywords:** POPs, PAHs, fish, GC-MS/MS, LC-MS/MS, clean-up, EMR-lipid

## Abstract

Interference of residual lipids is a very common problem in ultratrace analysis of contaminants in fatty matrices. Therefore, quick and effective clean-up techniques applicable to multiple groups of analytes are much needed. Cartridge and dispersive solid-phase extraction (SPE and dSPE) are often used for this purpose. In this context, we evaluated the lipid clean-up efficiency and performance of four commonly used sorbents—silica, C18, Z-Sep, and EMR-lipid—for the determination of organic pollutants in fatty fish samples (10%) extracted using ethyl acetate or the QuEChERS method. Namely, 17 polychlorinated biphenyls (PCBs), 22 organochlorine pesticides (OCPs), 13 brominated flame retardants (BFRs), 19 per- and polyfluoroalkyl substances (PFAS), and 16 polycyclic aromatic hydrocarbons (PAHs) were determined in this study. The clean-up efficiency was evaluated by direct analysis in real time coupled with time-of-flight mass spectrometry (DART-HRMS). The triacylglycerols (TAGs) content in the purified extracts were significantly reduced. The EMR-lipid sorbent was the most efficient of the dSPE sorbents used for the determination of POPs and PAHs in this study. The recoveries of the POPs and PAHs obtained by the validated QuEChERS method followed by the dSPE EMR-lipid sorbent ranged between 59 and 120%, with repeatabilities ranging between 2 and 23% and LOQs ranging between 0.02 and 1.50 µg·kg^−1^.

## 1. Introduction

The residues of ubiquitous environmental pollutants such as brominated flame retardants (BFRs), polychlorinated biphenyls (PCBs), organochlorine pesticides (OCPs), per-/polyfluoroalkyl substances (PFAS), or polycyclic aromatic hydrocarbons (PAHs) in food constitute a major concern in food safety programs [1,2,3]. For this reason, the availability of rapid, simple, robust, sensitive, and inexpensive analytical approaches is essential for the monitoring of these compounds to ensure that the food is safe for consumption. Occurrence data are also needed for the evaluation of dietary exposure and associated health risks [4].

Sample preparation is growing in importance in the analysis of complex food samples, especially those with a high lipid content, as the co-extracted matrix may have a detrimental impact on the method performance due to matrix effects. In this context, efficient sample purification is essential for unbiased identification and accurate quantification [5].

Due to the similar physico-chemical properties of PAHs, PCBs, OCPs, and BFRs, such as hydrophobicity (high log Kow values), relatively good thermal stability, and high boiling points, the ‘classical’ analytical methods for the determination of these compounds in various types of complex food matrices are often based on similar multi-step sample preparation procedures. These may include non-selective isolation of the lipid portion from the sample using non-polar solvents (*n*-hexane, dichloromethane, etc.), followed by various clean-up steps, using either destructive (e.g., sulphuric acid treatment or saponification) or non-destructive (GPC, SPE with different stationary phases) techniques and fractionations (for PAHs, saponification of lipids might be used as the first step prior to the extraction) [6,7,8,9,10,11,12,13,14]. The most commonly used sorbents in SPE are polar phases such as alumina, Florisil, silica, and/or their combination [5,15,16,17].

Where PFAS (which also have outstanding physico-chemical properties, such as high chemical and thermal stability, but, unlike other persistent organic pollutants (POPs), are amphiphilic) are concerned, methods based on solid-phase extraction (mainly using reverse phase C18 sorbent or ion change sorbents, WAX) or extraction with acetonitrile or methanol are usually employed [18,19]. Unfortunately, most of these methods are very laborious and require a relatively large amount of organic, often toxic solvents, such as dichloromethane, *n*-hexane, etc. [20,21].

Therefore, most procedures widely used for the isolation of these organic compounds in the last 10 years are based on the QuEChERS (Quick, Easy, Cheap, Effective, Rugged, and Safe) method, or some modification thereof. The modifications usually involve the extraction solvent, with ethyl acetate (EtOAc) being the most widely used for PAHs, PCBs, OCPs, and BFRs eligible for gas chromatography coupled to mass spectrometry (GC-MS) analysis [15,16,22,23]. Both solvents are considered environmentally friendly and therefore their use is very suitable for these methods [20,21]. To minimize the amount of potentially interfering matrix co-extracts present in the crude extracts, dispersive solid-phase extraction (dSPE) is widely employed. This simple and fast clean-up method is often used for the purification of acetonitrile extracts after the QuEChERS extraction. Various sorbents are used in dSPE (such as the primary secondary amine/PSA, C18, Z-Sep, EMR-lipid, charcoal, enviCarb, and their combinations) to remove not only lipids but also polar matrix co-extracts such as sugars, fatty acids, and other components. However, these sorbents sometimes fail to achieve adequate extract purification; in addition, they can exhibit nonselective interactions with analytes and cause unwanted analyte loss or strong matrix effects [15,24,25,26,27]. The final determination of PCBs, OCPs, polybrominated diphenyl ethers (PBDEs), and PAHs is typically performed using gas chromatography (GC) combined with (tandem) mass spectrometric detection (GC-MS/MS) [14,28,29,30]. On the other hand, liquid chromatography with (tandem) mass spectrometric detection (LC-MS/MS) is the method of a choice for the detection and quantitation of hexabromocyclododecane (HBCD) isomers, tetrabromobisphenol A (TBBPA), and PFAS [8,18,19,27,31]. However, there are some limitations in mass spectrometry due to matrix effects (ME), which can be particularly pronounced in complex matrices such as fish, where the analyte elutes together with other molecules, which without sufficient purification can lead to amplification or suppression of the analyte signal and can quantitatively alter the results of the analysis. For this reason, isotopically labeled internal standards are very often used to correct matrix effects [32,33,34].

Unfortunately, most methods for the determination of POPs are still optimized for only one group of contaminants, which leads to one sample being subsequently prepared in several ways for the determination of different groups of POPs. This approach is very inefficient because it consumes a lot of materials (especially solvents, many of which are toxic) and is time and money demanding. Due to the need to control food safety and lighten the environmental burden against different groups of POPs, sample preparation methods suitable for determining the broad spectrum of these contaminants are very much needed. Although analytes are determined in different ways (LC and GC analysis), it is necessary to find a sample preparation method that would be suitable for determining the broad spectrum of these substances.

The main objective of this study was to assess the efficiency of four different dSPE purification sorbents, routinely used for the clean-up of high fatty matrices, for the GC-MS/MS and LC-MS/MS determination of 71 POPs and 16 PAHs in a fish sample (10% lipid content) and to find a procedure that is most suitable for simultaneous determination of all the monitored groups of contaminants in fish.

## 2. Materials and Methods

### 2.1. Samples

A sample of skinless smoked trout fillet from the Czech retail market previously tested for the presence of OCPs, PCBs, PBDEs, PFAS, and PAHs was used for the experiments. Only detectable levels of BaA and CHR were measured in the test material. The concentrations were below the LOQs. The average lipid content of the test material was 10% (*w/w*). The sample was kept at −18 °C after homogenization.

### 2.2. Standards

PCB standards (all with declared purity of >97%), solid standards of OCPs, a standard mixture of 16 priority PAHs dissolved in cyclohexane (PAH Mix 9), and standards of individual PAHs, namely benzo[c]fluorene (BcFL), cyclopenta[*cd*]pyrene (CPP), 5-methylchrysene (5-MC), benzo[*j*]fluoranthene (BjFA), dibenzo[*a,l*]pyrene (DBalP), dibenzo[*a,e*]pyrene (DBaeP), dibenzo[*a,i*]pyrene (DBaiP), and dibenzo[*a,h*]pyrene (DBahP), also dissolved in cyclohexane, were purchased from Dr. Ehrenstorfer GmbH (declared purity ≥98%, Augsburg, Germany). Standard ^13^C-PCB 101, TBBPA standard and the isotopically labeled TBBPA internal standard, as well as a certified standard solution of ^13^C-labelled PAHs (US EPA 16 PAH Coctail), DBaiP-^13^C_12_, and DBaeP-^13^C_6_ were supplied by the Cambridge Isotope Laboratories (Andover, MA, USA). The individual standard solutions of the PBDE congeners dissolved in nonane, certified individual standards of the HBCD isomers (α, β, and γ) with their isotopically labeled internal standards, individual standards of PFAS, PFOS, as well as the isotopically labeled internal standards of PFAS were supplied by Wellington Laboratories (declared purity ≥ 98%, Guelph, ON, Canada). The PFOS standard contained 78.8% linear (linear perfluoro-1-octanesulfonate (L-PFOS)) and 21.2% branched isomers (branched perfluoro-1-octanesulfonates (Br-PFOS)), which allowed separate quantification of L- and Br-PFOS. Working standard solutions of HBCDs, TBBPA, and PFAS were prepared in methanol (MeOH) and stored in a refrigerator at 5 °C; the working standard solutions of PAHs, PCBs, PBDEs, and OCPs were prepared in isooctane (Iso) and stored at −20 °C. The calibration solutions concentration range was 0.05–100 ng mL^−1^.

### 2.3. Chemicals, Reagents, and Other Material

Methanol (MeOH), *n*-hexane (n-Hex), dichloromethane (DCM), and Iso were supplied by Merck (Darmstadt, Germany). Acetonitrile (MeCN), anhydrous magnesium sulfate (MgSO_4_), ethyl acetate (EtOAc), HPLC-grade ammonium acetate (99.99%), Z-Sep sorbent, and EMR-lipid (enhanced matrix removal-lipid) sorbent were obtained from Sigma Aldrich (Taufkirchen, Germany). All solvents were of analytical grade. Water purified using the Milli-Q Integral system supplied by Merck was used throughout the study. Sodium chloride (NaCl) was supplied by Lach-Ner (Neratovice, Czech Republic). Formic acid (85%) and ammonia solution (25%) were purchased from Penta (Chrudim, Czech Republic). Bondesil C18 sorbent (40 μm) was supplied by Varian (Harbor City, CA, USA). Silica (0.063–0.200 mm), supplied by Merck (Darmstadt, Germany), was activated by heating at 180 °C for 5 h, then deactivated by adding 2% of Milli-Q water and shaking for 3 h, and, finally, stored in a desiccator for 16 h before use. Anhydrous sodium sulfate (Na_2_SO_4_) obtained from Penta was heated at 600 °C for 5 h and then stored in a desiccator until use. Polypropylene (PP) 50 and 15 mL centrifuge tubes were supplied by Sigma-Aldrich (St. Louis, MI, USA). A Pasteur pipette (D812, 230 mm length) and glass wool were purchased from Poulten & Graf GmbH (Wertheim, Germany) and Merck (Kenilworth, NJ, USA), respectively.

### 2.4. Analytical Method

**Figure 1:**
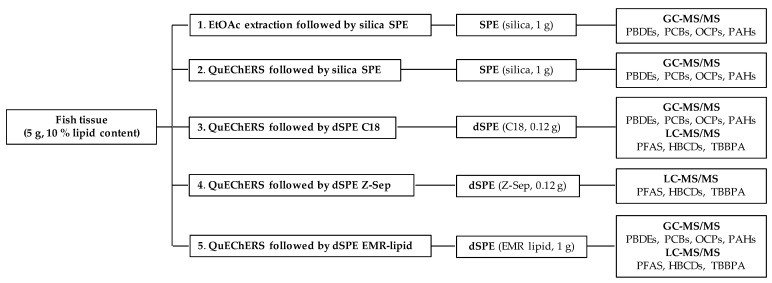
Flow charts of the tested methods.

Five sample preparation procedures were tested within this study. For clarity, all extraction methods described below are shown in Figure 1.

#### 2.4.1. EtOAc Extraction Followed by Silica SPE Clean-Up

For the EtOAc extraction followed by SPE cleanup, the method previously published by Kalachova et al. (2011) [17] was used. Briefly, 5 g of fish homogenate were weighed into a 50 mL falcon tube, mixed with 5 mL of Milli-Q water, and left to soak for 20 min. After that, 10 mL of EtOAc were added and the tube was shaken vigorously for 1 min. Subsequently, 4 g of anhydrous MgSO_4_ and 2 g of NaCl were added to the mixture. The tube was shaken for 1 min again and centrifuged for 5 min at 11,000× *g* (Hettich, Tuttlingen Germany). An aliquot of 5 mL from the EtOAc layer was taken from the tube and evaporated to the last drop of solvent. The crude extract was dissolved in 1 mL of *n*-hex and cleaned on a manually prepared silica SPE column (a Pasteur pipette filled with glass wool, 1 g of silica and approx. 0.2 g of Na_2_SO_4_). For the elution of the analytes, 10 mL of *n*-hex:DCM (3:1, *v/v*) was used. The cleaned-up extract was evaporated by a rotary vacuum evaporator (Büchi, Switzerland) and the rest of the solvents were removed using a gentle stream of nitrogen. The residue after evaporation was dissolved in 0.5 mL of Iso containing BDE 77 (5 ng.mL^−1^), ^13^C-PCB 101 (40 ng.mL^−1^), and ^13^C-labeled PAH (2 ng.mL^−1^) used as the syringe standard.

#### 2.4.2. QuEChERS Extraction Followed by Silica SPE-Clean-Up

The QuEChERS method was used for sample extraction. Briefly, 5 g of fish sample was mixed with 10 mL Milli-Q water in a 50 mL falcon tube and left to soak for 20 min. After the soaking, 10 mL of MeCN with 0.2 mL of formic acid were added and the mixture was shaken by hand for 1 min. Subsequently, 4 g MgSO_4_ and 1 g NaCl were added and the tube shaken for 1 min and centrifuged for 5 min at 11,000× *g*. The purification procedure was identical to that described in Section 2.4.1.

#### 2.4.3. QuEChERS Extraction Followed by dSPE C18 Clean-Up

The QuEChERS extraction was performed as described in Section 2.4.2. For the clean-up, an aliquot of 8 mL of the MeCN layer was transferred into a 15 mL falcon tube containing 0.12 g C18 and 1.2 g MgSO_4_. The tube was shaken for 1 min and centrifuged for 5 min at 11,000× *g*. An aliquot of 5 mL from the MeCN layer was filtered through a 0.2 μm nylon centrifuge tube filter and evaporated to the last drop of solvent. Each extraction was performed in duplicates and the resulting residues after evaporation were dissolved in either 0.5 mL of MeOH for LC-MS/MS analysis or in 0.5 mL of Iso containing BDE 77 (5 ng.mL^−1^), ^13^C-PCB 101 (40 ng.mL^−1^), and ^13^C-labeled PAH for GC-MS/MS analysis.

#### 2.4.4. QuEChERS Extraction Followed by dSPE Z-Sep Clean-Up

The sample was processed as described in Section 2.4.3 but the dSPE sorbent Z-Sep (0.12 g/8 mL) was used for the purification of crude extract. The residue after evaporation was dissolved in 0.5 mL of MeOH for LC-MS/MS analysis.

#### 2.4.5. QuEChERS Extraction Followed by dSPE EMR-Lipid Clean-Up

The sample was processed according to the optimized method described in the Application note No. 5991-6088EN [35]. A total of 5 g of the fish sample was weighed into a 50-mL PP centrifuge tube, mixed with 10 mL MeCN and vigorously shaken by hand for 1 min. Subsequently, 4 g anhydrous MgSO_4_ and 1 g NaCl were added to the tube, the tube was vigorously shaken for 1 min again, and centrifuged for 5 min at 11,000× *g*. An aliquot of 8 mL of the organic layer was transferred into a 50-mL PP tube containing 6 mL of Milli-Q water and 1 g of the EMR-lipid sorbent. The tube was shaken for 1 min and centrifuged for 5 min at 11,000× *g*. In the next step, an aliquot of 12 mL was transferred to the tube containing 6 g anhydrous MgSO_4_ and 1.5 g NaCl. The tube was shaken for another 1 min and centrifuged again for 5 min at 11,000× *g*. An aliquot of 5 mL from the MeCN layer was evaporated to the last drop of solvent. Each extraction was performed in duplicates and the resulting residues after evaporation were dissolved in either 0.5 mL of MeOH for LC-MS/MS analysis or in 0.5 mL of Iso containing BDE 77 (5 ng.mL^−1^), ^13^C-PCB 101 (40 ng.mL^−1^), and ^13^C-labeled PAH for GC-MS/MS analysis.

#### 2.4.6. Gravimetrical Determination of Co-Extracts

For the gravimetric determination, 5 mL of the extracts obtained as described above were used. The extracts were pipetted into pre-weighed 10-mL flasks and the solvent was evaporated to dryness. The amount of co-extracts was determined by differential weighing.

#### 2.4.7. DART-HRMS Detection of Co-Extracted Lipids

Ambient mass spectrometry based on the Direct Analysis in Real Time (DART) ion source coupled to an Exactive^TM^ benchtop high-resolution mass spectrometer with orbitrap mass analyzer (Thermo Fisher Scientific, Bremen, Germany) was used for rapid determination and identification of matrix co-extracts. The method used to identify the matrix co-extracts has been previously described by Hrbek et al. [36]. Briefly, the DART-HRMS was operated in both the positive and negative ionization mode. The optimized parameters settings were as follows: (i) DART ionization—helium flow: 2.5 L min^−1^; gas temperature: 400 °C; discharge needle voltage: −5000 V; grid electrode: ±350 V; and (ii) mass spectrometric detection: capillary voltage: ±50 V, tube lens voltage: ±120 V; capillary temperature: 250 °C. The mass resolving power calculated for *m/z* 200 of the mass spectrometer was 50,000 fwhm (full width at half maximum). The mass spectra acquisition rate was 2 spectra s^−1^. The mass spectra were recorded in the range *m/z* 50–1100. Ammonia was used as a support dopant. The obtained mass spectral data were background-subtracted in the Xcalibur software (version 2.1, Thermo Fisher Scientific, San Jose, CA, USA), which was also used for the estimation of ion elemental composition. SIMCA software (v. 13.0, Umetrics, Umea, Sweden) was employed for the chemometric analysis.

#### 2.4.8. GC-MS/MS Determination of PCBs, PBDEs, OCPs, and PAHs

A gas chromatograph Agilent 7890A coupled to a triple quadrupole mass spectrometer Agilent 7000B (Agilent Technologies, Palo Alto, CA, USA) operated in the electron ionization mode (EI) was used for the instrumental measurements of this group of contaminants. The GC system was equipped with a programmable temperature vaporization (PTV) injector. The GC conditions were as follows: oven temperature programme: Rxi^®^-PAH (40 m × 0.18 mm i.d. × 0.07 µm; Restek, PA, USA): 50 °C (2.9 min); 30 °C.min^−1^ to 240 °C; 2 °C.min^−1^ to 270 °C; and 40 °C.min^−1^ to 340 °C (12 min). Helium was used as the carrier gas, flow rate: 1.3 mL.min^−1^; PTV injection: mode solvent vent; injection volume: 1 × 8 μL; initial temperature of inlet: 50 °C (0.17 min); inlet rating velocity: 600 °C; and final inlet temperature: 325 °C. The MS detector interface temperature was set at 280 °C, the quadrupole temperature at 150 °C, and the ion source temperature at 280 °C. The tandem mass spectrometer was operated in the multiple reaction monitoring (MRM) mode detecting at least two transitions per analyte. An overview of both quantitative and confirmation MS/MS transitions and collision energies (CE selected) for each compound in the EI mode is summarized in Table S1 (Supplementary Materials). GC-MS/MS data were evaluated using the MassHunter Workstation Software (v. B07.00, Agilent Technologies, Palo Alto, CA, USA).

#### 2.4.9. LC-MS/MS Determination of PFAS, HBCDs, and TBBPA

The UHPLC analyses of PFAS, HBCDs, and TBBPA were performed using the Acquity Ultra-Performance LC system (Waters, Milford, MA, USA). For the determination of the target compound, the method published by Lankova et al. [37] was used. For the separation, an Acquity UPLC HSST 3 analytical column (100 mm × 2.1 mm i.d., 1.8 μm particle size, Waters) maintained at 40 °C was used. The mobile phase consisted of (A) 5 mM ammonium acetate in Milli-Q water; and (B) MeOH. The gradient elution under the following conditions was used: 10–50% B over 0.5 min, then 50–100% B over 7.5 min, followed by an isocratic hold at 100% B for 4 min. The flow rate began at 0.3 mL.min^−1^ and simultaneously with the linear gradient the flow rate change from 0.3 to 0.4 mL.min^−1^. The injected sample volume was 5 µL. The LC system was coupled to a tandem quadrupole mass spectrometer Xevo TQ-S (Waters) equipped with electrospray ion source operated in negative-ion mode. The ion source settings were as follows: needle potential −4500 V, curtain gas 25 psi, nebulizer (Gas 1) and Turbo gas (Gas 2) 55 psi, temperature of Turbo gas 650 °C. As well as in GC-MS/MS, the analytes were determined in MRM mode with two transitions per analyte. The quantitative and qualitative MRM transitions of the target analytes are listed in the Table S1 in the Supplementary Materials. The MassLynx and Multiquant software packages were used for the LC-MS/MS measurement evaluation.

### 2.5. Quality Assurance/Quality Control

The performance characteristics (recovery, repeatability, and LOQ) of the tested methods were obtained by the analysis of six replicates (at a single spiking level) of the smoked trout (fat content 10%) sample. The matrix was spiked with the standard mixture of 71 POPs and 16 PAHs analytes at different levels, which were processed as described in Section 2.5. The spiking level was 5 µg·kg^−1^ for PCBs and OCPs, 2 µg·kg^−1^ for PBDEs and PAHs, 0.25 µg·kg^−1^ for PFAS, and 2.5 µg·kg^−1^ for HBCDs and TBBPA. Recoveries were calculated as the ratio of the measured concentration and the amount of the respective compound added to these samples before the extraction. Repeatability was expressed as the relative standard deviation (RSD; %). LOQs were estimated as the lowest calibration standard with the signal-to-noise ratio (S/N) > 10 for the quantitative transition (ion), and S/N > 3 for at least one confirmation transition (ion). For the lowest calibration levels, matrix calibration standards were prepared. To prevent possible matrix effects, isotopically labeled analytes for quantitation of PAHs, BFRs, and PFAS were used.

## 3. Results and Discussion

As mentioned in the Introduction, a sample preparation procedure including efficient clean-up is a critical condition for a reliable GC-MS/MS as well as LC-MS/MS analysis of various environmental contaminants occurring in food matrices. It is worth mentioning that the character of the adverse effects of the co-extracted matrix on the performance may differ between these two techniques. The objective of this study was to assess the potential of various types of sorbents (silica, C18, Z-Sep, and EMR-lipid) for introducing a uniform sample clean-up step suitable for rapid analysis of the contaminants suitable for both GC-MS/MS (PCBs, PBDEs, OCPs, and PAHs) and LC-MS/MS (PFAS, HBCDs, and TBBPA) analysis. Smoked trout representing a high-fat matrix was used for testing.

The comparison of the tested methods is summarized and critically evaluated in the following paragraphs. In total, five different methods often used for the determination of POPs and PAHs in fatty matrices, such as fish, were compared: (1) EtOAc extraction followed by silica SPE clean-up; (2) QuEChERS extraction followed by silica SPE clean-up; (3) QuEChERS extraction followed by dSPE C18 clean-up; (4) QuEChERS extraction followed by dSPE Z-Sep clean-up; and (5) QuEChERS extraction followed by dSPE EMR-lipid clean-up.

### 3.1. Comparison of the Clean-Up Efficiency

**Figure 2:**
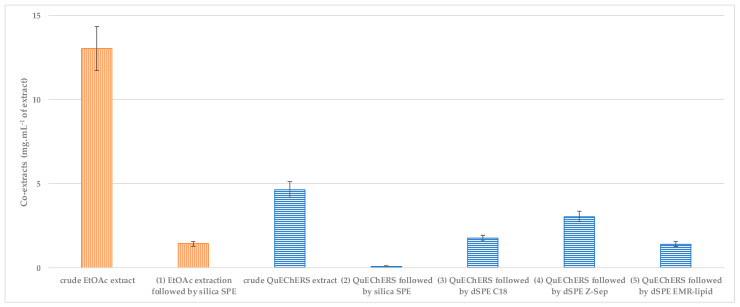
The amount of matrix co-extracts determined gravimetrically in smoked trout extracts before and after purification employing various sorbents for SPE and dSPE. Error bars display the repeatability of the procedure.

The efficiency for the removal of co-extracts was in the first stage estimated by gravimetrical determination of the residue after evaporating the solvent from the purified extract; in the next step, the character of the co-extracts was investigated by the DART-HRMS technique. The results of the gravimetrical determination are summarized in Figure 2.

As shown in Figure 2, the crude EtOAc extract contained almost three times more co-extracts than the crude QuEChERS extract. The purification efficiency was determined as the reduction in co-extracts content after purification of the respective crude extracts using SPE or dSPE (crude EtOAc and QuEChERS extract = 100%).

As expected, silica SPE exhibited the best efficiency (98%) in removing non-polar matrix co-extracts from the QuEChERS extract. A good purification efficiency of 89% was achieved by the same sorbent also in the case of the EtOAc extract, despite the fact that the amount of extracted lipids was more than two times higher. Unfortunately, this approach is very labor-intensive and does not support high laboratory throughput. In this case, the preparation of 10 samples took approximately 2 h of laboratory work. The purification of the QuEChERS extract achieved with the dSPE EMR-lipid sorbent showed quite promising results—with a purification efficiency of 70%, the amount of residual co-extracts was comparable to that achieved by the EtOAc extraction in combination with silica SPE clean-up. On the other hand, the poorest extract clean-up efficiency was observed for the Z-Sep dSPE sorbent (purification efficiency 35%).

**Figure 3:**
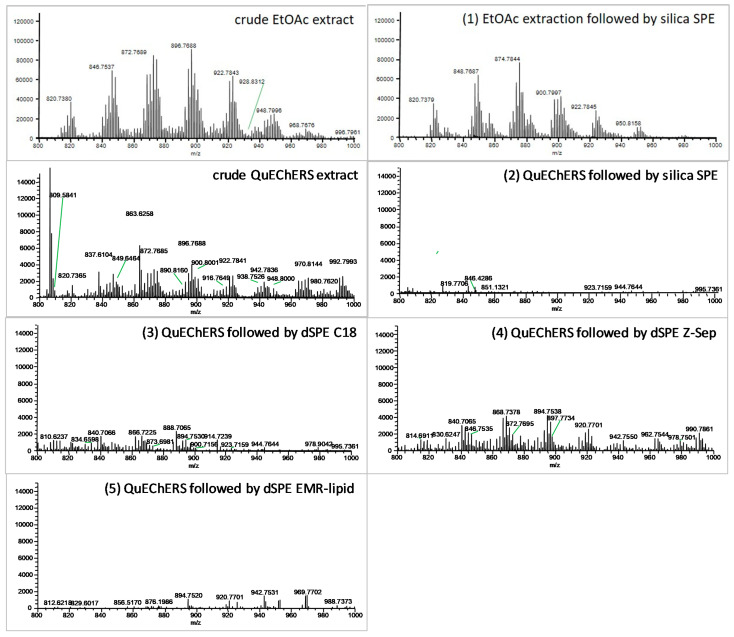
The comparison of TAG removal (scanned mass range m/z 800–1000) between the crude and purified extracts of the smoked trout using different sorbents detected by DART-HRMS.

The DART-HRMS technique was employed for a rapid inspection of the purified extracts. Mass spectra of the various extracts collected at the mass range of m/z 800–1000 are presented in Figure 3. DART(+) ammonia adducts of the molecular ions [M+NH_4_]^+^ of TAG and the respective deacylated fragment ions dominated the spectra.

In line with the gravimetrical analysis, a significant decrease in the TAGs content was observed in samples after the QuEChERS extraction cleaned using the silica SPE and dSPE using the EMR-lipid sorbent. The lowest dSPE clean-up potential was observed for the Z-Sep sorbent. The same trend as for TAGs was also identified for fatty acids (see Figure S1). As the amount of co-extracts was relatively high, the working characteristics of the monitored substances were not further investigated for this procedure using GC-MS/MS.

### 3.2. Verification Study

Once the cleaning efficiency of the sorbents was established, the verification of the selected methods was performed, and their working characteristics were determined and compared. The obtained methods’ performance characteristics are shown in Table 1, Tables S2 and S3 (Supplementary Materials).

It must be noted that the results achieved for PFAS, TBBPA, and HBCDs using dSPE clean-up were not compared with those obtained by silica clean-up due to a higher affinity of these analytes to the stationary phase (silica and Florisil), resulting in low recoveries [18,19].

Considering the results obtained for the GC amenable contaminants (PCBs, PBDEs, OCPs, and PAHs) for which silica purification can be used, it is evident that lower recoveries were obtained for the method employing MeCN as the extraction solvent. This result was expected because EtOAc, due to its lower polarity, is more suitable for the extraction of these less polar substances. In line with a previous study published by Kalachova et al. [17], the most problematic in terms of recoveries was the group of OCPs for which significant losses during purification with silica SPE of dieldrin, endrin, β-endosulfan, and endosulfan sulphate were observed. The recoveries of these compounds were in the range of 5–36% only, and, therefore, this method of extract purification is not suitable for these substances. Regarding repeatabilities, for both tested solvents (EtOAC and MeCN) comparable results were measured and the repeatabilities were in the range 1–19%. Although the recoveries of MeCN extraction were lower, this approach still met the requirements for method validation: method yield 70–120% and repeatability < 20%, except for dieldrin, endrin, β-endosulfan, and endosulfan sulphate.

When comparing the performance characteristics of the QuEChERS extraction combined with SPE/dSPE cleanup for analytes determined by GC-MS/MS, similar results were obtained after purification with silica and the EMR-lipid sorbent (Table 1). The recoveries of the EMR-lipid method ranged between 71 and 97%, with repeatabilities ranging between 3 and 14% for PCBs, PBDEs, PAHs, and most of the OCPs. Lower recoveries were obtained for dieldrin, *α-, β-*, and *γ-*HCH, and oxychlordane (38–50%). In the case of the C18 sorbent, a significant reduction in recoveries for PAHs (59–86%) and some OCPs (see Table S2) was observed. The Z-Sep sorbent was not tested for these analytes due to the high amount of co-extracts, as discussed in detail in Section 3.1.

If we evaluate the methods from the point of view of their toxicological and exposure hazards, it should be noted that more environmentally friendly is the extraction using ethyl acetate than the MeCN extraction, because the total analytical hazard values (taHV) of EtOAc is 7.3 while MeCN is 26.8. In the case of both solvents, the purification step employing DCM and *n*-hex, significantly increases their environmental score. In the case of EtOAc extraction followed by purification on the silica column, the procedure hazard value (pHV) is 1696, and for MeCN extraction followed by silica SPE, the pHV is 1891 [21]. For this reason, it is also advisable to choose a more environmentally friendly sample purification procedure, such as dSPE. The pHV calculated for the QuEChERS method followed by dSPE is 268, which is 7 times less than for the QuEChERS method followed by silica SPE. Thus, we can conclude that the methods using dSPE are significantly more environmentally friendly compared to approaches with other purifications, where it is necessary to change solvents, such as SPE.

**Figure 4:**
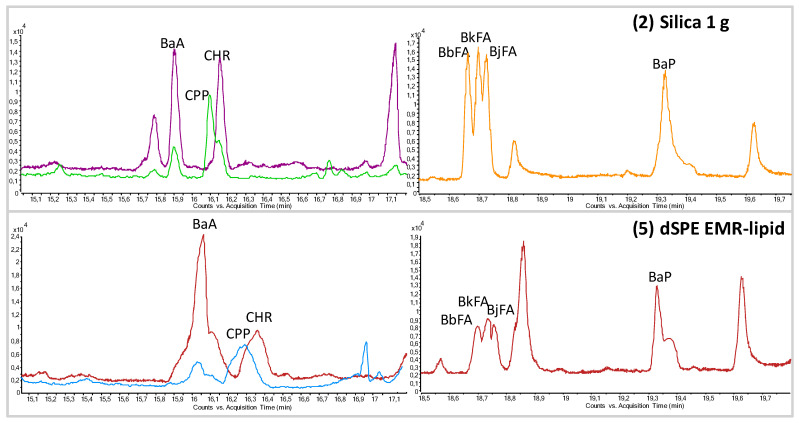
An example of the GC-MS/MS chromatogram in multi reaction monitoring (MRM) mode of a smoked trout sample spiked with PAHs. MeCN extract purified using (**2**) silica SPE minicolumn and (**5**) dSPE EMR-lipid. MRM transitions shown: CPP-226 > 226; BaA, CHR-228 > 228, BbFA, BkFA, BjFA, BaP-252 > 252.

In GC-MS/MS analysis, some problems were encountered, mainly concerning PAHs (see Figure 4). As shown in the chromatogram, there were some co-elutions with co-extracts, which lead to two-fold increases in the LOQ and worsening the peak shapes. To achieve lower LOQs and avoid other problems associated with co-extracts, an additional clean-up step, such as silica SPE, is needed for removing TAGs and other co-extracts. No problem with the matrix co-extracts and peak shapes were observed for the remaining POP groups analyzed by GC-MS/MS, as shown in Figure 4. The sample chromatograms of the other groups of POPs are shown in Figures S2 and S3.

**Figure 5:**
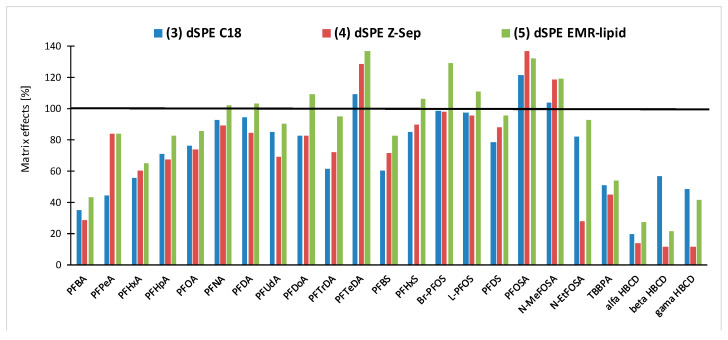
Matrix effects using the tested sorbents (C18, Z-Sep and EMR-lipid) on LC-MS/MS. Response 100% = no matrix effect; response < 100% = signal suppression (e.g., response 40% corresponds to 60% suppression of signal by matrix effects); and response > 100% = signal enhancement.

Where HBCDs, TBBPA, and PFAS, for which only the dSPE clean-up was tested (see above), are concerned, comparable results were obtained using all the tested sorbents for PFAS. Slightly lower recoveries (49–57%) were obtained for HBCDs when the C18 was used for clean-up. As a part of the validation study, matrix effects on the LC-MS/MS analysis for PFAS, HBCDs, and TBBPA were also investigated and C18, Z-Sep, and EMR-lipid clean-ups were compared from this perspective. The matrix effects were assessed using a comparison of the matrix match calibration standard with the solvent standard. As shown in Figure 5, the lowest signal suppression was obtained by the acetonitrile extraction followed by dSPE EMR-lipid. A generally higher signal suppression for early eluting PFAS (C4-C6 PFCA) was observed. Furthermore, a significant response suppression, up to 90%, was found for HBCDs and TBBPA, and, therefore, matrix calibration or labeled internal standards were necessary for quantification.

## 4. Conclusions

In this study, five different sample preparation strategies for determining 71 POPs and 16 PAHs in smoked trout representing a fatty matrix (fat content 10%) were tested. The effectiveness of the sample clean-up was compared on the basis of TAG removal, evaluated using the DART-HRMS technique. Silica SPE and dSPE EMR-lipid performed the best in removing these co-extracted interfering matrix components. The performance characteristics (recovery, repeatability, and LOQ) were obtained for most of the tested procedures, and in case of LC-MS/MS analysis (PFSA, HBCDs and TBBPA), matrix effects were also evaluated. The clean-up sorbent EMR-lipid was applicable for the determination of the majority of the target POPs in fatty fish, with the exceptions of dieldrin, *α-, β-, γ-*HCH, and oxychlordane due to their low recoveries (<50%). Recoveries achieved by this clean-up procedure were within the range of 71–97% and repeatabilities were <14%. Although a significant decrease in TAGs was observed after purification using EMR-lipid, the removal of the interfering co-extracted matrix components was not sufficient for the determination of PAHs using GC-MS/MS. This was due to the high ‘chemical noise’ at retention times where the target analytes are eluted, which led to an increase in LOQs. For the analysis of PAHs, therefore, additional optimization of the purification step is needed. Although the method using the EMR sorbent for extract purification will increase the LOQs (2–5 times) for most of the analytes monitored, these LOQs are still low enough to be able to use this method to determine POPs in food for legislative control and for food safety and environmental exposure monitoring.

## Figures and Tables

**Table 1:** Method performance characteristics obtained in the verification study (REC—recovery (%); RSD—repeatability (%); LOQ—limit of quantification).

		GC-MS/MS				LC-MS/MS		
		PAHs	PCBs	PBDEs	OCPs	PFASs	HBCD	TBBPA
**Spiking concentration**	**(µg·kg^−1^)**	2	5	5	2	0.25	2.5	2.5
**Recovery %**	**EtOAc + silica SPE**	81–104	83–119	89–117	5–108	-	-	
	**QuEChERS + silica SPE**	70–91	76–110	77–92	9–103	-	-	
	**QuEChERS + C18**	59–86	74–110	76–101	65–114	88–130	49–57	73
	**QuEChERS + Z-Sep**	-	-	-	-	85–116	65–116	102
	**QuEChERS + EMR-lipid**	71–97	74–88	73–94	38–92	94–120	65–91	76
**RSD %**	**EtOAc + silica SPE**	1–15	3–19	3–18	1–11	-	-	
	**QuEChERS + silica SPE**	3–15	3–12	4–12	2–13	-	-	
	**QuEChERS + C18**	4–12	6–13	5–10	3–19	1–9	2–5	2
	**QuEChERS + Z-Sep**	-	-	-	-	1–10	1–2	1
	**QuEChERS + EMR-lipid**	3–13	4–13	5–12	4–14	2–11	10–23	6
**LOQ (µg·kg^−1^)**	**EtOAc + silica SPE**	0.05–0.25	0.10–0.50	0.50	0.1–0.5	-	-	
	**QuEChERS + silica SPE**	0.05–0.25	0.10–0.50	0.50	0.1–0.5	-	-	
	**QuEChERS + C18**	0.05–0.25	0.10–0.50	0.50	0.1–0.5	0.01–0.06	0.30	0.30
	**QuEChERS + Z-Sep**	-	-	-	-	0.01–0.06	0.30	0.30
	**QuEChERS + EMR-lipid**	0.10–0.50	0.10–0.50	0.25–0.50	0.50	0.02–0.50	1.50	1.50

Min–max. RSD %—relative standard deviation (*n* = 6).

## Supplementary Materials

The following are available online at https://www.mdpi.com/article/10.3390/toxics10010012/s1. Table S1: MRM transitions. Table S2: Method performance characteristic obtained using GC-MS/MS. Table S3: Method performance characteristic obtained using LC-MS/MS. Figure S1: The comparison of fatty acids removal (scanned mass range m/z 100–500) between crude and purified extracts of the smoked trout using different sorbents detected by DART(-)-HRMS. Figure S2: GC-MS/MS MRM chromatograms of target OCPs, PCBs and PBDEs in spiked smoked trout (concentration level: PAHs, OCPs = 2 µg·kg^−1^; PCBs, PBDEs = 5 µg·kg^−1^, MRM transitions are shown in Table S1). Figure S3: LC-MS/MS MRM chromatograms of smoked trout spiked with (A) HBCDs, TBBPA and (B) PFAS (concentration level: PFAS–0.25 µg·kg^−1^ and HBCD and TBBPA = 2.5 µg·kg^−1^, MRM transitions are shown in Table S1).
