# Supplementary material for: Critical Assessment of Clean-Up Techniques Employed in Simultaneous Analysis of Persistent Organic Pollutants and Polycyclic Aromatic Hydrocarbons in Fatty Samples
Source: Toxics. 2022 Jan 1;10(1):12. doi: 10.3390/toxics10010012 (PMC8781265; doi:10.3390/toxics10010012)
Supplement: Supplementary file 1 [file toxics-10-00012-s001.zip › toxics-1511989-supplementary.pdf]

toxics

Supplementary Materials

Critical Assessment of Clean-Up Techniques Employed in Simultaneous Analysis of Persistent Organic Pollutants and Polycyclic Aromatic Hydrocarbons in Fatty Samples

Lucie Drábová, Darina Dvořáková, Kateřina Urbancová, Tomáš Gramblička, Jana Hajšlová and Jana Pulkrabová *

Table S1. MRM transitions.

Group	Analyte	Quantification MRM transition (CE)*				Confirmation MRM transition (CE)*				Confirmation MRM transition (CE)*			
	CB 28	255.8	>	186	32	255.8	>	151	50	257.8	>	186	34
	CB 52	291.8	>	222	34	291.8	>	257	12	291.8	>	220	34
	CB 77	289.9	>	220	24	291.9	>	222	27	-	-	-	-
	CB 81	289.9	>	220	25	291.9	>	220	24	-	-	-	-
	CB 101	325.9	>	256	34	325.9	>	291	12	325.9	>	254	34
	CB 105	326.9	>	256	27	325.9	>	291	12	325.9	>	254	27
	CB 114	327.9	>	256	24	323.9	>	254	24	-	-	-	-
	CB 118	326	>	256	27	326	>	254	27	-	-	-	-
**PCBs**	CB 123	325.9	>	256	25	323.9	>	254	25	-	-	-	-
	CB 126	326.9	>	256	27	325.9	>	291	12	325.9	>	254	27
	CB 138	361.7	>	290	30	359.8	>	325	15	359.8	>	290	30
	CB 153	361.7	>	290	35	359.8	>	325	16	359.8	>	290	35
	CB 156	359.8	>	290	24	357.8	>	287.9	25	-	-	-	-
	CB 167	359.8	>	290	25	357.8	>	287.9	24	-	-	-	-
	CB 169	360	>	290	27	357.8	>	287.9	24	-	-	-	-
	CB 180	373.7	>	324	34	391.8	>	321.9	28	395.7	>	326	34
	CB 189	391.8	>	321.9	34	393.7	>	359	15	395.7	>	325	34
	Aldrin	257	>	222	12	263	>	191	30	263	>	193	30
	chlordane *cis*	373	>	266	25	373	>	337	4	373	>	264	25
	chlordane *trans*	373	>	266	25	373	>	337	4	373	>	264	25
	dieldrin	263	>	193	30	263	>	191	30	-	-	-	-
	endosulfan *alfa*	241	>	206	15	195	>	159	5	229	>	194	10
	endosulfan *beta*	195	>	159	5	229	>	194	10	239	>	204	15
	endosulfan *sulphate*	272	>	237	20	387	>	217	5	387	>	253	5
	Endrin	263	>	193	35	263	>	191	35	281	>	245	20
	HCB	284	>	214	35	284	>	249	10	284	>	142	50
	HCH *alfa*	219	>	183	10	219	>	145	20	181	>	109	15
**OCPs**	HCH *beta*	181	>	145	15	181	>	109	30	219	>	145	10
	HCH *gama*	219	>	183	5	181	>	109	12	181	>	145	12
	heptachlor	272	>	237	25	272	>	217	40	274	>	239	20
	heptachlor epoxid *exo*	353	>	263	25	353	>	282	20	253	>	218	40
	heptachlor epoxid *endo*	183	>	155	10	353	>	282	15	353	>	263	30
	DDD, *o,p′-*	235	>	165	20	235	>	200	8	235	>	199	15
	DDD, *p,p′-*	235	>	165	20	235	>	200	8	235	>	199	15
	DDE, o,p'-	246	>	176	30	246	>	211	20	248	>	176	30
	DDE, *p,p′-*	246	>	176	30	246	>	211	20	248	>	176	30
	DDT, *o,p′-*	235	>	165	20	235	>	200	8	237	>	165	20
	DDT, *p,p′-*	235	>	165	20	235	>	200	8	237	>	165	20

Group	Analyte	Quantification MRM transition (CE)*				Confirmation MRM transition (CE)*				Confirmation MRM transition (CE)*			
	oxychlordane	185	>	149	4	187	>	151	4	387	>	263	14
	BDE 28	405.8	>	246	20	407.8	>	248.1	22	-	-	-	-
	BDE 47	485.7	>	326	28	483.7	>	324	32	-	-	-	-
	BDE 49	485.7	>	326	28	483.7	>	324	32	-	-	-	-
	BDE 85	565.7	>	405.8	28	403.8	>	269.9	35	-	-	-	-
	BDE 99	565.7	>	405.8	28	403.8	>	269.9	35	-	-	-	-
	BDE 100	565.7	>	405.8	28	403.8	>	269.9	35	-	-	-	-
**BFRs**	BDE 153	643.6	>	483.8	20	483.7	>	374.9	40	-	-	-	-
	BDE 154	643.6	>	483.8	20	483.7	>	374.9	40	-	-	-	-
	BDE 183	561.7	>	454.9	45	721.6	>	561.8	17	-	-	-	-
	TBBPA	541		446	32	541		418	44				
	α-HBCD	640,7	>	80,9	16	640,7	>	78,9	16				
	β-HBCD	640,7	>	80,9	16	640,7	>	78,9	16				
	γ-HBCD	640,7	>	80,9	16	640,7	>	78,9	16				
	5-MCH	242	>	242	5	242	>	241	19	-	-	-	-
	BaA	228	>	228	5	228	>	226	38	-	-	-	-
	BaP	252	>	252	5	252	>	250	44	-	-	-	-
	BbFA	252	>	252	5	252	>	250	44	-	-	-	-
	BcFL	216	>	216	5	216	>	215	19	-	-	-	-
	BghiP	276	>	276	10	276	>	274	50	-	-	-	-
	BjFA	252	>	252	5	252	>	250	44	-	-	-	-
	BkFA	252	>	252	5	252	>	250	44	-	-	-	-
	CHR	228	>	228	5	228	>	226	38	-	-	-	-
	CPP	226	>	226	5	226	>	224	44	-	-	-	-
	DBahA	278	>	278	5	278	>	276	52	-	-	-	-
	DBaeP	302.1	>	302.1	15	302.1	>	300.1	40	-	-	-	-
**PAHs**	DBahP	302.1	>	302.1	15	302.1	>	300.1	40	-	-	-	-
	DBaiP	302.1	>	302.1	15	302.1	>	300.1	40	-	-	-	-
	DBalP	302.1	>	302.1	15	302.1	>	300.1	40	-	-	-	-
	IP	276	>	276	10	276	>	274	50	-	-	-	-
	BaA- $^{13}C_6$	233.9	>	231.9	38	233.9	>	233.9	5	-	-	-	-
	BaP- $^{13}C_4$	255.9	>	255.9	5	255.9	>	253.9	44	-	-	-	-
	BbFA- $^{13}C_6$	258	>	258	5	258	>	256	44	-	-	-	-
	BghiP- $^{13}C_{12}$	288	>	288	10	288	>	286	50	-	-	-	-
	BkFA- $^{13}C_6$	258	>	258	5	258	>	256	44	-	-	-	-
	CHR- $^{13}C_6$	233.9	>	233.9	5	233.9	>	231.9	38	-	-	-	-
	DBahA- $^{13}C_6$	284	>	284	5	282	>	282	5	-	-	-	-
	DBaeP- $^{13}C_6$	308	>	308	15	308	>	306	40	-	-	-	-
	DBaiP- $^{13}C_{12}$	314	>	314	15	314	>	312	40	-	-	-	-
	IP- $^{13}C_6$	282	>	282	10	282	>	280	50	-	-	-	-
	PFBA	213	>	169	8								
	PFPeA	263	>	219	8								
	PFHxA	313	>	269	8	313	>	119	20				
	PFHpA	363	>	319	4	363	>	169	16				
	PFOA	413	>	369	8	413	>	219	16	413	>	169	20
	PFNA	463	>	419	8	463	>	219	16	463	>	169	20
**PFAS**	PFDA	513	>	469	8	513	>	269	16	513	>	219	16
	PFUdA	563	>	519	8	563	>	269	16				
	PFDoA	613	>	569	12	613	>	319	20				
	PFTrDA	663	>	619	12	663	>	319	16				
	PFTeDA	713	>	669	12	713	>	219	24				
	PFBS	299	>	99	36	299	>	80	36				

Group	Analyte	Quantification MRM transition (CE)*				Confirmation MRM transition (CE)*				Confirmation MRM transition (CE)*
	PFHxS	399	>	99	40	399	>	80	40	
	Br-PFOS	499	>	99	48	499	>	80	64	
	L-PFOS	499	>	99	48	499	>	80	64	
	PFDS	599	>	98,9	52	599	>	79,9	80	
	PFOSA	498	>	478	24	498	>	78	40	
	N-MeFOSA	512	>	269	24	512	>	219	28	
	N-EtFOSA	526	>	269	28	526	>	219	24	

Table S2. Method performance characteristic obtained using GC-MS/MS.

	GC-MSMS											
	EtOAc extraction followed by silica SPE			QuEChERS followed by silica SPE			QuEChERS followed by dSPE C18			QuEChERS followed by dSPE EMR-lipid		
	REC %	RSD %	LOQ (μg/kg)	REC %	RSD %	LOQ (μg/kg)	REC %	RSD %	LOQ (μg/kg)	REC %	RSD %	LOQ (μg/kg)
aldrin	80	2	0.30	80	4	0.30	66	18	0.30	78	13	0.50
DDD, *o,p′*-	103	4	0.50	103	5	0.50	79	3	0.50	88	6	0.50
DDD, *p,p′*-	98	2	0.50	98	2	0.50	77	3	0.50	84	9	0.50
DDE, o,p'-	97	5	0.50	82	5	0.50	75	3	0.50	79	4	0.50
DDE, *p,p′*-	95	3	0.50	85	6	0.50	72	3	0.50	81	6	0.50
DDT, *o,p′*-	96	3	0.50	89	8	0.50	79	3	0.50	92	5	0.50
DDT, *p,p′*-	97	1	0.50	87	4	0.50	77	3	0.50	90	4	0.50
dieldrin	*36-*	*15*	*0.50*	*32*	*6*	*0.50*	107	9	0.50	41	12	0.50
endosulfan *alpha*	81	4	0.50	77	5	0.50	92	12	0.50	79	4	0.50
endosulfan *beta*	*26*	*19*	*0.50*	*24*	*8*	*0.50*	92	13	0.50	80	8	0.50
endosulfan *sulfate*	*5*	*16*	*0.50*	*9*	*12*	*0.50*	114	11	0.50	81	7	0.50
endrin	*28*	*8*	*0.50*	*26*	*9*	*0.50*	82	16	0.50	75	10	0.50
heptachlor	82	2	0.30	79	8	0.30	72	17	0.30	82	9	0.50
HEPO (endo) *trans*	83	4	0.30	80	6	0.30	86	12	0.30	81	12	0.50
HEPO (exo) *cis*	86	5	0.30	78	5	0.30	86	10	0.30	80	14	0.50
hexachlorobenzene	87	10	0.10	76	5	0.10	65	4	0.10	74	6	0.50
HCH *alfa*	94	2	0.10	84	4	0.10	83	7	0.10	*42*	*8*	*0.50*
HCH *beta*	108	11	0.10	79	8	0.10	84	6	0.10	*46*	*8*	*0.50*
HCH *gama*	88	5	0.10	75	4	0.10	84	6	0.10	*50*	*12*	*0.50*
chlordane *cis*	96	4	0.50	87	5	0.50	88	19	0.50	72	9	0.50
chlordane *trans*	94	1	0.50	84	3	0.50	85	10	0.50	77	11	0.50
oxychlordane	81	2	0.50	79	13	0.50	78	10	0.50	*38*	*10*	*0.50*
PCB28	105	4	0.10	84	7	0.10	84	8	0.10	81	8	0.10
PCB52	119	9	0.10	110	4	0.10	90	6	0.10	76	9	0.10
PCB 77	95	9	0.25	83	8	0.25	74	10	0.25	82	4	0.25
PCB 81	102	7	0.25	81	5	0.25	82	7	0.25	82	7	0.25
PCB101	107	19	0.10	98	12	0.10	80	7	0.10	85	11	0.10
PCB 105	93	6	0.10	76	8	0.10	82	9	0.10	79	4	0.10
PCB 114	97	6	0.10	77	7	0.10	79	13	0.10	75	8	0.10
PCB118	88	7	0.10	85	9	0.10	76	8	0.10	83	5	0.10
PCB 123	98	6	0.10	78	4	0.10	85	6	0.10	78	4	0.10
PCB 126	87	3	0.10	87	6	0.10	110	9	0.10	88	9	0.10
PCB138	98	5	0.10	88	8	0.10	84	8	0.10	86	13	0.25
PCB153	97	4	0.10	77	5	0.10	92	8	0.10	85	12	0.25
PCB 156	88	6	0.10	78	7	0.10	88	6	0.10	78	6	0.25
PCB 167	92	4	0.10	82	3	0.10	81	9	0.10	74	9	0.25
PCB 169	83	5	0.10	79	5	0.10	79	10	0.10	81	5	0.25
PCB 180	86	4	0.50	76	9	0.50	80	7	0.50	75	10	0.50
PCB 189	87	5	0.50	77	4	0.50	76	6	0.50	78	7	0.50
PBDE 28	92	11	0.50	80	6	0.50	76	8	0.50	82	14	0.25

GC-MSMS												
	EtOAc extraction followed by silica SPE			QuEChERS followed by silica SPE			QuEChERS followed by dSPE C18			QuEChERS followed by dSPE EMR-lipid		
	REC %	RSD %	LOQ (µg/kg)	REC %	RSD %	LOQ (µg/kg)	REC %	RSD %	LOQ (µg/kg)	REC %	RSD %	LOQ (µg/kg)
PBDE 47	94	5	0.50	78	5	0.50	80	8	0.50	82	6	0.25
PBDE 49	95	3	0.50	91	4	0.50	76	5	0.50	74	9	0.25
PBDE85	117	18	0.50	92	11	0.50	85	7	0.50	87	6	0.25
PBDE 99	100	9	0.50	81	7	0.50	86	6	0.50	73	7	0.50
PBDE 100	89	6	0.50	79	7	0.50	81	8	0.50	80	7	0.50
PBDE 153	90	10	0.50	80	12	0.50	101	10	0.50	92	8	0.50
PBDE154	97	8	0.50	77	5	0.50	80	9	0.50	84	5	0.50
PBDE 183	96	12	0.50	83	10	0.50	78	7	0.50	94	12	0.50
5MC	86	2	0.05	74	3	0.05	74	9	0.05	77	13	0.25
BaA	97	4	0.05	91	5	0.05	69	5	0.05	83	5	0.10
BaP	92	3	0.05	81	4	0.05	75	6	0.05	81	9	0.10
BbFA	85	3	0.05	79	3	0.05	81	8	0.05	75	11	0.10
BcFL	103	4	0.05	75	6	0.05	74	5	0.05	71	3	0.25
BghiP	101	1	0.05	72	8	0.05	70	7	0.05	75	5	0.25
BjFA	97	4	0.05	70	5	0.05	75	5	0.05	97	3	0.10
BkFA	97	5	0.05	82	3	0.05	77	6	0.05	95	9	0.10
CPP	86	5	0.05	84	4	0.05	59	4	0.05	86	8	0.10
DBaeP	92	3	0.25	79	6	0.25	68	10	0.25	89	6	0.50
DBahA	82	2	0.05	86	4	0.05	72	8	0.05	83	11	0.10
DBahP	81	15	0.25	82	10	0.25	70	10	0.25	82	6	0.50
DBaiP	96	3	0.25	78	8	0.25	65	12	0.25	72	8	0.50
DBalP	93	4	0.25	74	5	0.25	75	12	0.25	83	8	0.50
CHR	104	3	0.05	83	5	0.05	86	8	0.05	77	8	0.10
IP	87	12	0.05	76	15	0.05	79	6	0.05	89	6	0.10

Table S3. Method performance characteristic obtained using LC-MS/MS.

LC-MSMS									
	QuEChERS followed by dSPE C18			QuEChERS followed by dSPE Z-Sep			QuEChERS followed by dSPE EMR-lipid		
	REC %	RSD %	LOQ (µg/kg)	REC %	RSD %	LOQ (µg/kg)	REC %	RSD %	LOQ (µg/kg)
PFBA	107	9	0.06	97	10	0.06	107	4	0.50
PFPeA	102	4	0.06	99	7	0.06	112	4	0.10
PFHxA	105	2	0.02	105	5	0.02	120	11	0.10
PFHpA	106	3	0.01	106	2	0.01	109	2	0.05
PFOA	106	1	0.01	109	1	0.01	108	4	0.05
PFNA	112	1	0.01	107	1	0.01	106	3	0.05
PFDA	107	1	0.01	105	1	0.01	110	3	0.05
PFUdA	105	1	0.01	106	1	0.01	102	4	0.05
PFDoA	111	1	0.01	107	1	0.01	109	3	0.05
PFTrDA	130	1	0.01	116	1	0.01	112	5	0.05
PFTeDA	127	1	0.01	110	1	0.01	109	5	0.05
PFBS	99	1	0.01	102	1	0.01	96	3	0.05
PFHxS	102	1	0.01	105	1	0.01	110	3	0.05
Br-PFOS	122	1	0.02	112	1	0.01	109	4	0.02
L-PFOS	127	1	0.01	114	1	0.01	108	3	0.04
PFDS	105	1	0.01	107	1	0.01	102	4	0.05
PFOSA	88	1	0.01	88	1	0.01	95	4	0.05
N-MeFOSA	120	1	0.01	85	2	0.01	109	9	0.05
N-EtFOSA	96	1	0.01	85	1	0.01	94	5	0.05
TBBPA	73	2	0.30	102	1	0.30	76	6	1.50
α-HBCD	49	5	0.30	116	1	0.30	91	23	1.50

	LC-MSMS								
	**QuEChERS followed by dSPE C18**			**QuEChERS followed by dSPE Z-Sep**			**QuEChERS followed by dSPE EMR-lipid**		
	**REC %**	**RSD %**	**LOQ (μg/kg)**	**REC %**	**RSD %**	**LOQ (μg/kg)**	**REC %**	**RSD %**	**LOQ (μg/kg)**
β-HBCD	57	2	0.30	103	1	0.30	70	18	1.50
γ-HBCD	49	2	0.30	65	2	0.30	65	10	1.50

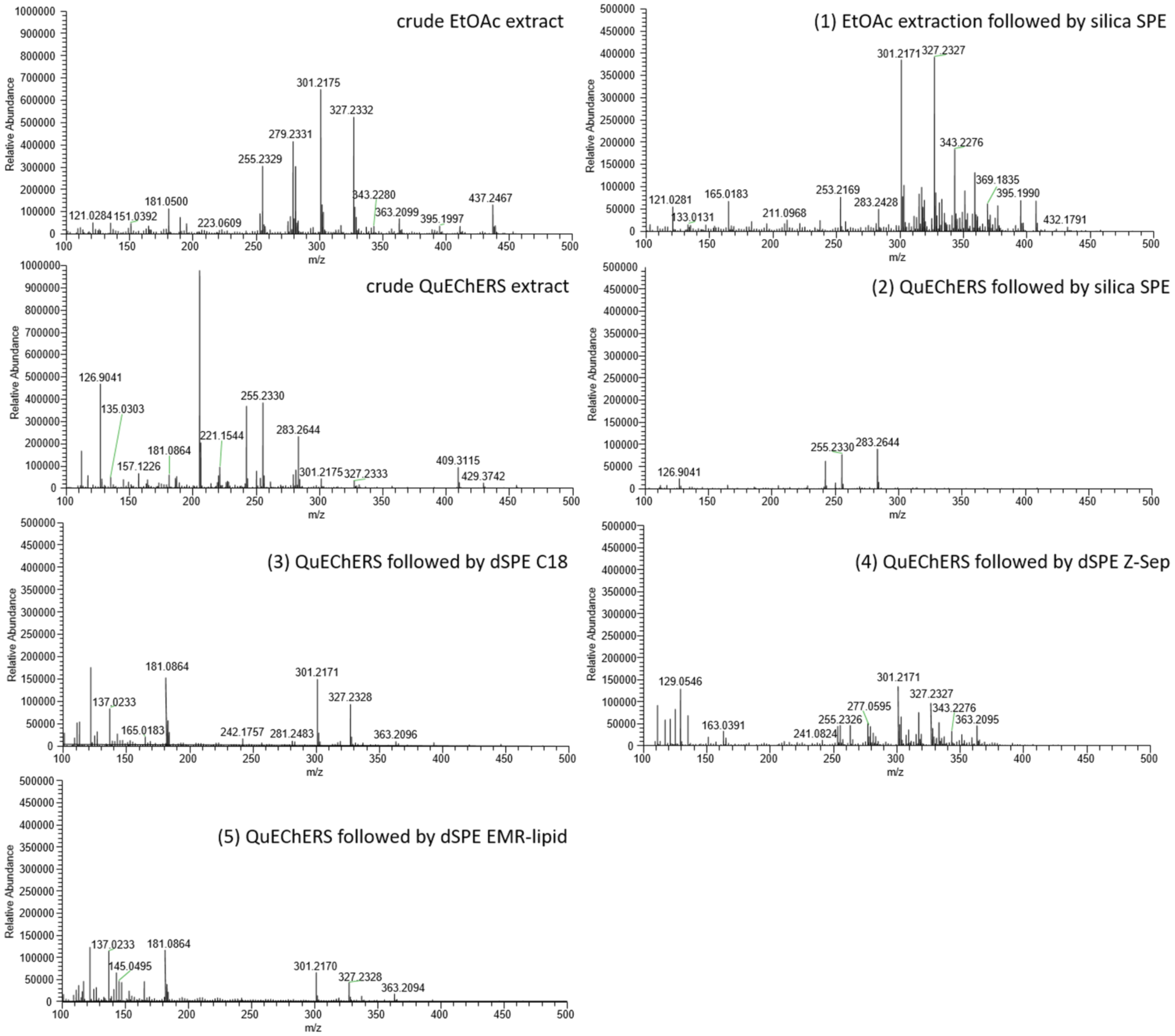

Figure S1. The comparison of fatty acids removal (scanned mass range *m*/*z* 100–500) between crude and purified extracts of the smoked trout using different sorbents detected by DART(-)-HRMS.

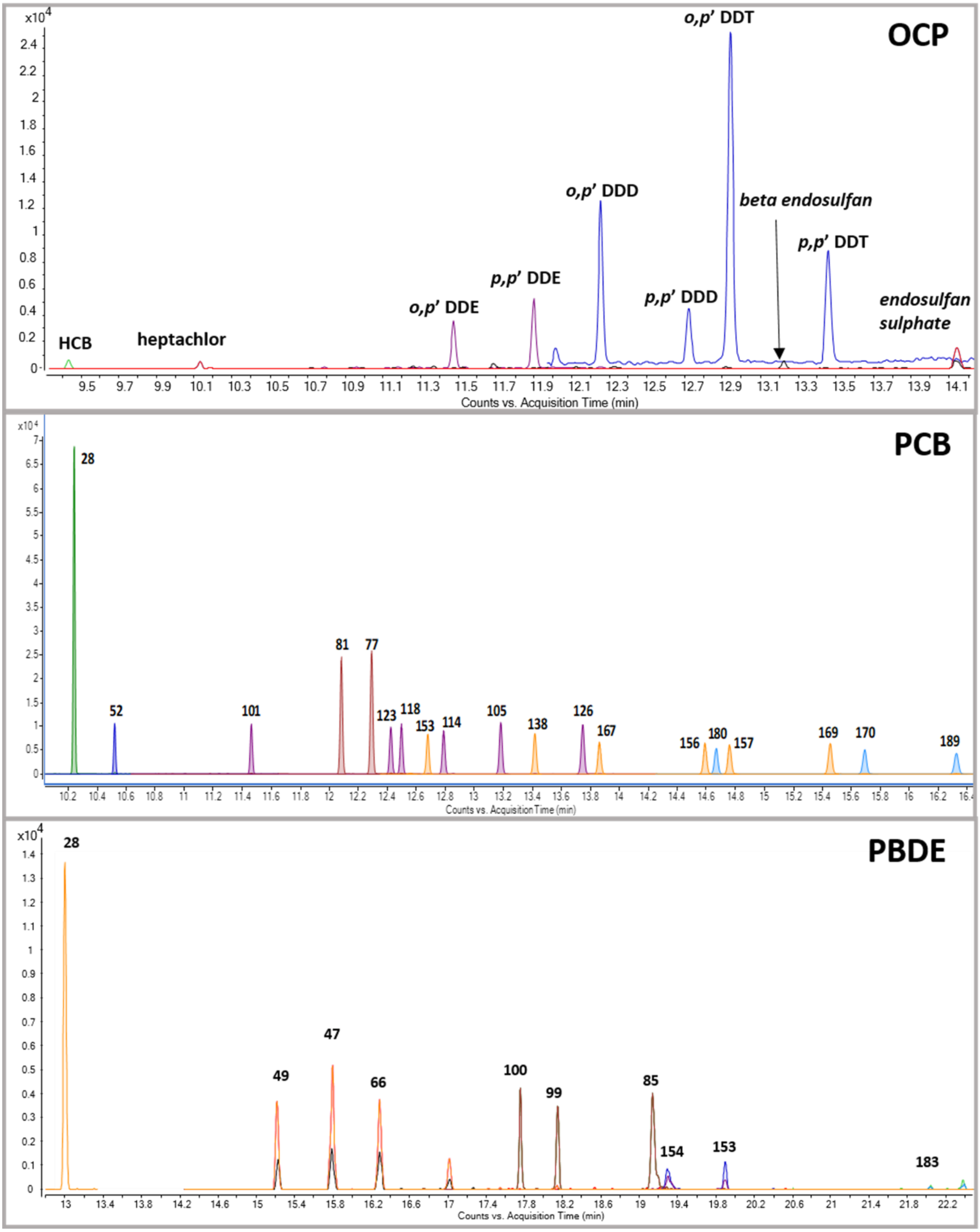

Figure S2. GC-MS/MS MRM chromatograms of target OCPs, PCBs and PBDEs in spiked smoked trout (concentration level: PAHs, OCPs = 2 µg.kg^{-1}; PCBs, PBDEs = 5 µg.kg^{-1}, MRM transitions are shown in Table S1).

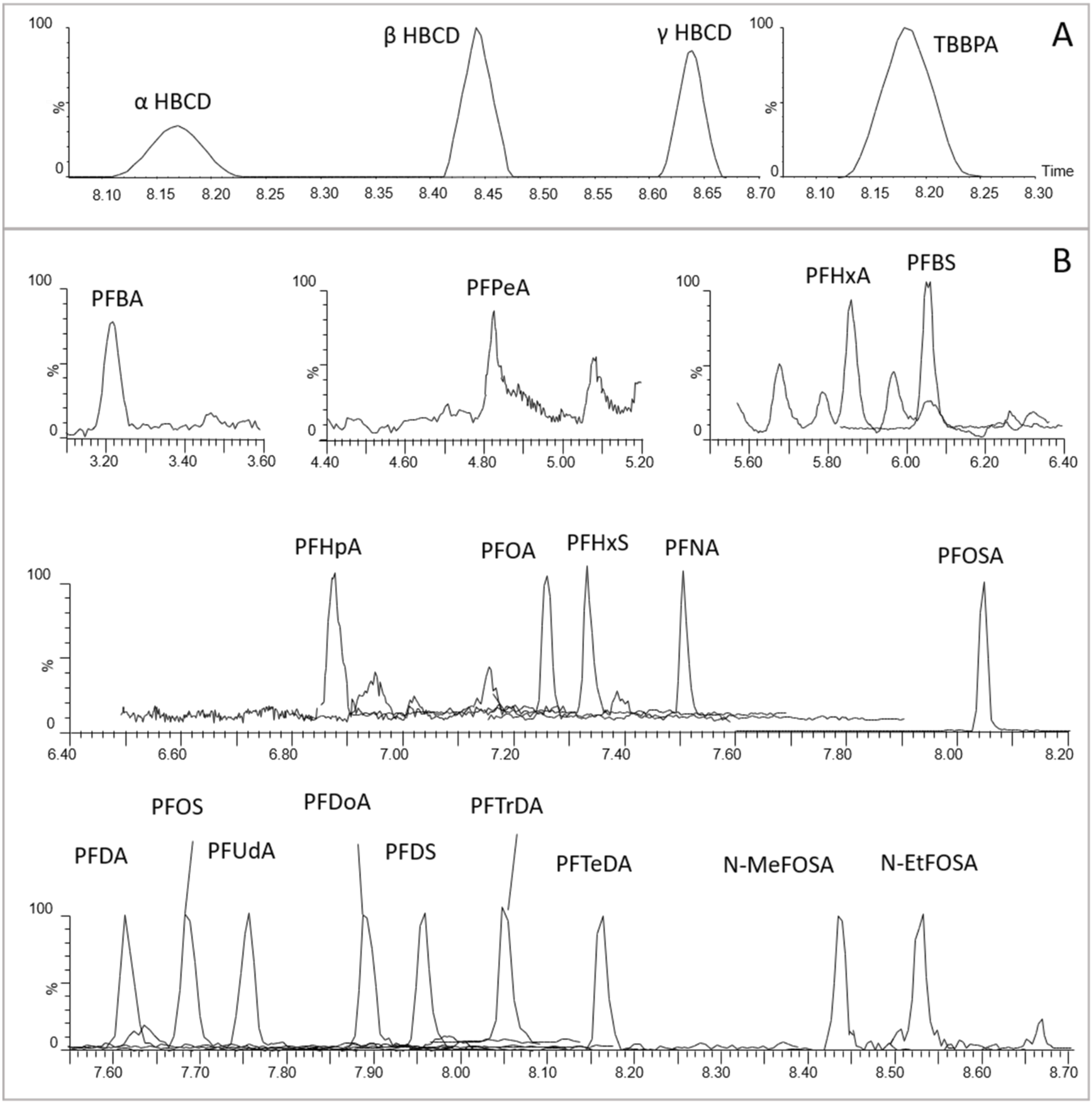

Figure S3. LC-MS/MS MRM chromatograms of smoked trout spiked with (A) HBCDs, TBBPA and (B) PFAS (concentration level: PFAS–0.25 μg.kg^{-1} and HBCD and TBBPA = 2.5 μg.kg^{-1}, MRM transitions are shown in Table S1).
